# Health in Harmony: Integrating Community Strengths to Bridge Disparities

**DOI:** 10.1089/heq.2024.0198

**Published:** 2025-04-10

**Authors:** Lauren Anderson, Caison Black, Rochelle H. Holm, Michael O. Emerson, Ted Smith

**Affiliations:** ^1^ Center for Healthy Air Water and Soil, Christina Lee Brown Envirome Institute, School of Medicine, University of Louisville, Louisville, Kentucky, USA.; ^2^ Baker Institute for Public Policy, Rice University, Houston, Texas, USA.

**Keywords:** built environment, environmental health, health equity, health disparity, institutional racism, model

## Abstract

There is a need to shift from deficit-based environmental health approaches toward a more balanced framework that also considers community strengths at a granular level. The Universal Basic Neighborhood Framework integrates both qualitative and quantitative data across 35 health-supportive elements within topics of environmental, housing, social, and transportation domains, promoting an understanding of health as emerging from a range of environmental factors. The framework provides a more balanced approach by presenting both negative and positive health determinants, promotes leveraging community strengths and voice in public health interventions, and enables better understanding of community health needs and assets.

## Introduction

The shift from focusing solely on health care to recognizing the importance of social determinants of health marks an advancement in public health, broadening the understanding of the diverse factors shaping individual and community well-being.^1^ In the United States, the social determinants of health are operationalized through tools such as the County Health Rankings, which aim to mobilize collective action around community health by establishing broad responsibility across fields such as education, housing, and health care.^2^ However, affluent white communities set the standards when health outcomes are ranked in this manner.^3^ In addition, relative ranking can obscure health disparities and their root causes for certain communities. For instance, in the two areas investigated for this study, although County Health rankings show that Louisville, KY, has 1 dentist per 940 people, the metric masks stark differences in dental care utilization across neighborhoods; 37% of residents of Area 1 visited a dentist in the past year compared with 70% of residents from Area 2.^4,5^

**FIG. 1.**
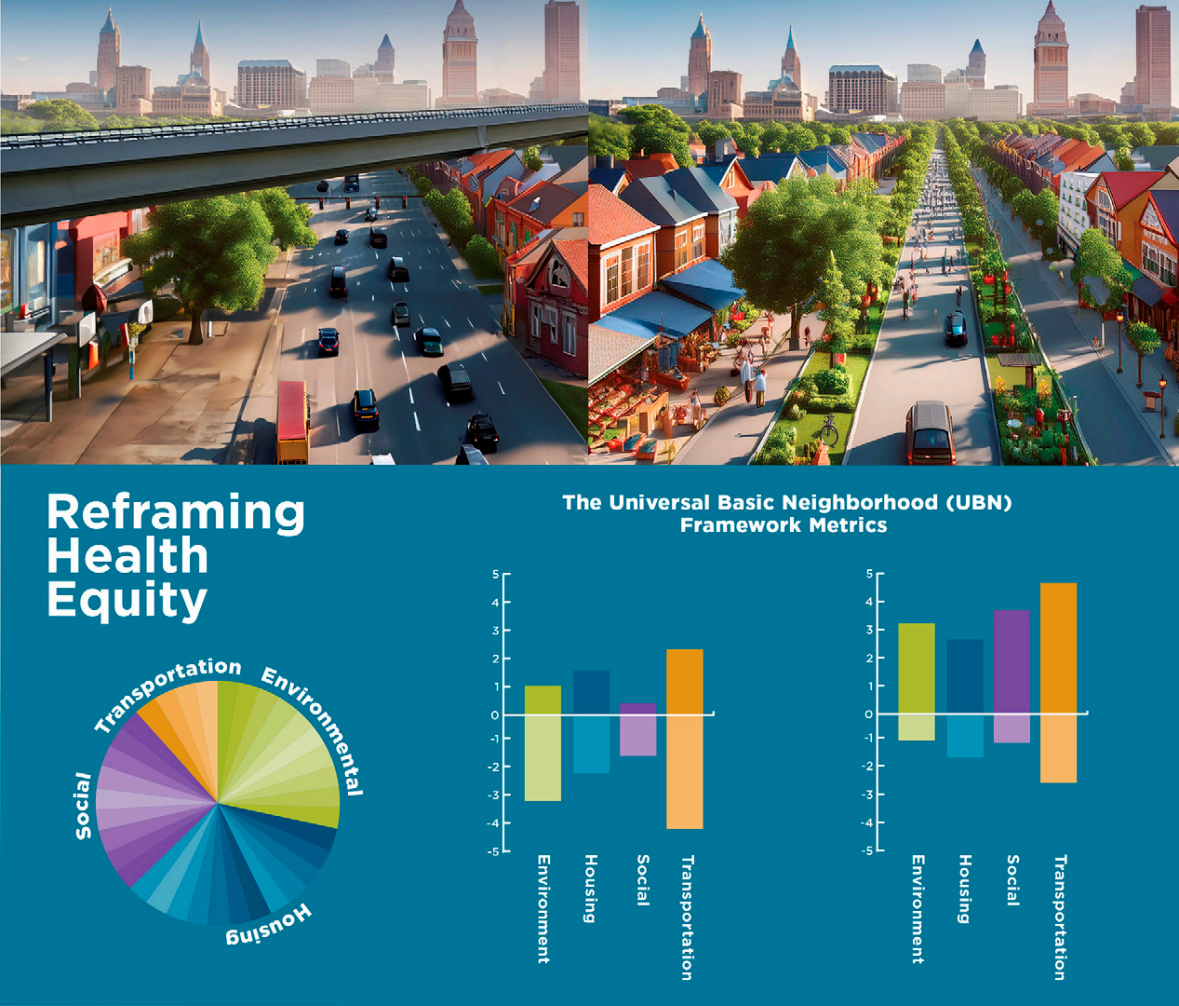
Framework for integrating both negative and positive health determinants to bridge neighborhood disparities. The universal basic neighborhood framework includes 35 place-based characteristics to define place-based health.

The United States has mandated certain essential assets such as clean drinking water and basic trash and sanitation services. However, multiple factors that support good health have not been uniformly prioritized or protected. For example, although long-term improvements to the built environment promote health, health equity efforts have been impeded by a preference for short-term outcomes and a focus on disease treatment.^6,7^ Furthermore, narrow definitions of health, biases toward specific groups, an overemphasis on certain populations at the expense of others, and limited attention to mechanisms that drive health disparities can limit progress.^8^ New methodologies are required to capture the complexity of intersecting identities, social gradients in health, and community-level strengths (Fig. 1). Inspired by the ideas of guaranteed income and salutary environments, the Universal Basic Neighborhood (UBN) framework offers a method to identify and assess neighborhood characteristics that are essential for health. To expand the scope of health determinants, this framework includes 35 place-based health-associated characteristics. This approach shifts away from comparing neighborhoods and instead emphasizes each area's capacity for improvement. By centering on universal health, the UBN framework offers a new vision that prioritizes every community’s full potential for well-being.

## Health Data and Relative Rankings: Falling Short of What We Need

Administrative data gathered by government agencies, health care providers, and other organizations during routine activities have been useful for describing the general population, but not subpopulation, health baselines.^9^ Covering large populations over long periods, these data allow robust statistical analysis and generalizable health patterns. The UBN framework leverages these strengths, while addressing four major weaknesses. First, administrative data miss key social variables, leading to a focus on clinical outcomes and health care usage at the expense of the underlying causes of health disparities. Overlooking factors, such as social cohesion, support networks, historical discrimination, and access to resources, can result in a narrow focus on disease treatment rather than prevention and health promotion. Second, administrative datasets at large scales lack the granularity necessary to address neighborhood-level health needs and environmental conditions in marginalized and underserved communities.^9^ Communities of color and individuals with intersecting or marginalized identities are at the greatest risk of being lost from big data.^10^ Third, administrative data lack a qualitative context. The absence of community-specific insights, experiences, and impacts of social factors can exaggerate the disconnect between data and realized health outcomes, potentially leading to policies and interventions mismatched to on-the-ground realities. Finally, health is often analyzed using relative rankings, which can engender bias, overemphasize deficits, obscure important health dimensions, and obfuscate protective factors. The relative ranking results in the most advantaged groups, representing an ideal outcome threshold. This creates a biased perception that affluent, predominantly white, areas represent ideal health standards, whereas a large portion of the U.S. population has not met such thresholds.^3^ Furthermore, the deficit of assets, such as grocery stores, is emphasized without exploring the potential protective value of abundant grocery options. Focusing on deficits leads to the pathologizing of communities, reinforcing that certain places are inherently “unhealthy,” rather than acknowledging the constraints of place-based factors on the ability of individuals to achieve health. This deficit-focused view diminishes the strengths, resilience, and protective factors of communities, such as social cohesion, family networks, and cultural practices that promote well-being. The focus on data deficits can be considered institutional racism, by which racial inequities in social determinants of health develop and persist.^11^ To move beyond this, we must rethink how we design and support these environments, ensuring every place has minimum acceptable standards for health-promoting resources, infrastructure, and services.

## UBN Framework

Place is critical in shaping health outcomes, influencing everything from acute and chronic diseases to overall well-being. Neighborhoods have different housing, transportation, and social and environmental qualities that can be positive (supportive of health) or negative (introducing health risks). Therefore, our UBN framework prioritizes place-based strategies, ensuring that interventions are rooted in the local context and leverage unique community assets. The UBN framework integrates place-based characteristics (assets and liabilities), qualitative data, and community input and sets positive, neutral, and negative thresholds for each metric. Positive and neutral thresholds are required in addition to health equity conversations because, although negative thresholds are clearly defined for environmental quality, such as air and noise pollution exposure, mirrored positive scaling for beneficial qualities is lacking. For example, meeting minimum air quality standards does not equate to “healthy air,” but rather that pollution levels do not pose unreasonable risk to health. In contrast, meeting a positive minimum tree canopy standard of 20%–30% is likely to confer health-supportive benefits to that community and equate to a healthier place.^12^ Our framework thus emphasizes reducing risks such as pollution and noise exposure and incorporating positive thresholds, such as green spaces and social cohesion, as essential elements of a healthy community.

Drawing from global literature on urban design and healthy cities, the proposed UBN framework integrates 35 factors known to impact health (Table 1).^2,13–21^ Environmental factors such as urban heat, noise pollution, air pollution, and access to nature impact health by contributing to stress, respiratory and cardiovascular issues, and mental well-being, with harmful conditions exacerbating illness, whereas access to natural environments promotes physical and emotional health.^22,23^ Housing factors such as affordable housing, secure tenure, and access to grocery stores, childcare, hospitals, and well-maintained areas can reduce stress and support physical, mental, and social well-being.^24^ Transportation factors such as an accessible public transportation network, alternative transportation usage, and vehicle availability impact health by reducing traffic-related air pollution, promoting physical activity, and decreasing stress.^25^ Social factors, such as social service and cultural centers, social cohesion and connection, economic equality, and safety foster belonging, reduce stress, enhance mental well-being, and promote overall health and resilience.^21^

**Table 1.** A Better Environmental Health Model: The Universal Basic Neighborhood Framework (*N* = 35)

Factor	Metric (*n* = 35)
Environmental factors (*n* = 10)	
Urban heat	Average maximum warm season temperature
Noise pollution	Average 24-hour exposure to noise pollution
Air pollution	Average 24-hour particulate matter (PM2.5) concentration
	Toxic emission hazards
	Traffic Proximity Index
Pollution sources	Deteriorated (Lead) Paint Index
	Point sources (Superfund sites, cement plants, metal recyclers, oil refineries, power generation stations, incinerators, etc.)
Exposure to nature	Park access
	Park area per capita
	Tree canopy coverage
Housing factors (*n* = 12)	
Housing cost	Affordable housing
	Housing security
	Public housing available
	Secure tenure
	Subsidized housing rate
	Utility security
Housing context	Access to internet
	Adequate childcare
	Area cleanliness
	Grocery access
	Proximity to jobs (commute length)
	Proximity to acute care hospitals
Transportation factors (*n* = 4)	
Public transportation	Accessible public transport network
	Alternative transportation usage
Vehicle availability	Vehicle availability
Road safety	Traffic fatalities and serious injury per capita
Social factors (*n* = 9)	
Community assets	Cultural outlets per capita
	Social service centers
Social supports	Diversity Index
	Social connection
	Social support
Economic equality	Gini Index
	Low Poverty Index
Civic engagement	Voter registration
Community safety	Low prevalence of crime

**FIG. 2.**
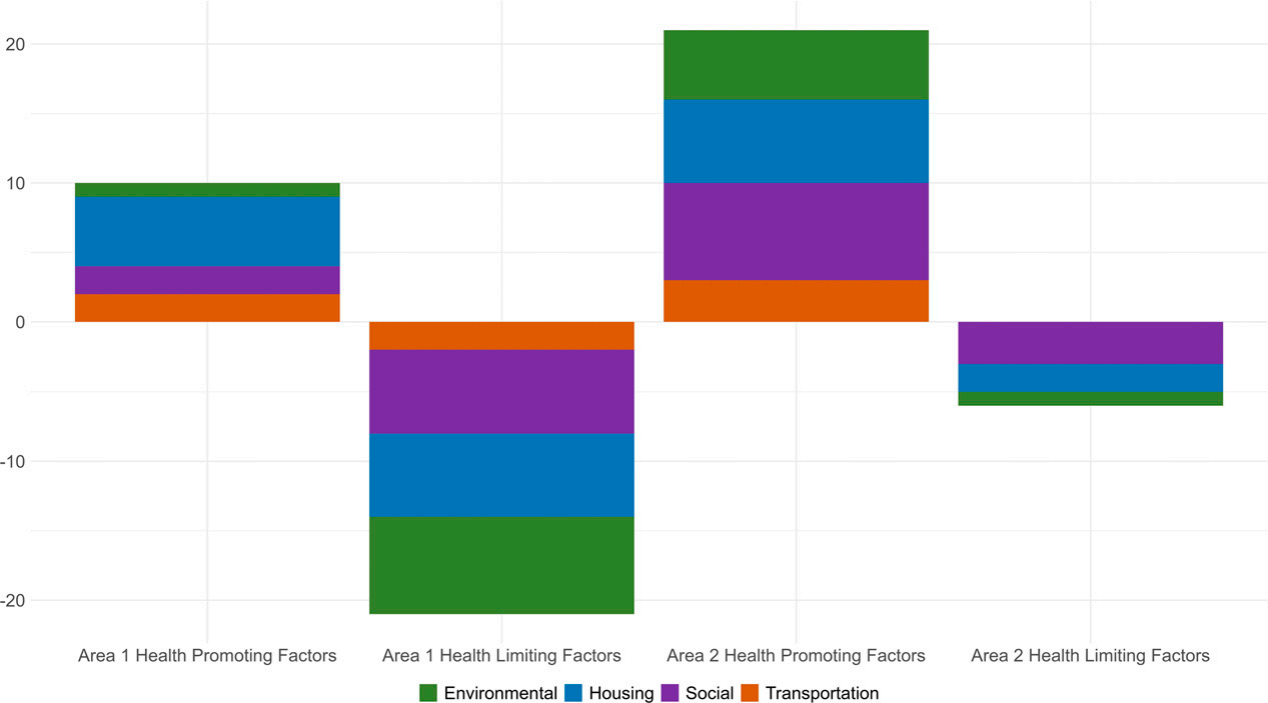
Results from evaluating two neighborhoods for environmental health using the Universal Basic Neighborhood framework methodology. Area 1 contained 10 positive health-promoting and 21 health-limiting factors. Area 2 had 21 health-promoting and 6 health-limiting factors.

To test the framework, we examined the degree to which these factors were present or absent in two demographically and socioeconomically diverse neighborhoods in Louisville, KY. For each metric, a positive, neutral, or negative score was applied and tallied across the domains (Fig. 2). Area 1 contained 10 positive health-promoting and 21 health-limiting factors. Area 2 had 21 health-promoting and 6 health-limiting factors. The assessment uncovered strengths in all four factor areas (environmental, housing, transportation, and social) and surprising assets, such as strong park access in Area 1.

## Future Directions

This framework helps prioritize areas for health interventions, as leaning into park improvements in Area 1 may yield more impactful outcomes than park improvements in Area 2. It also identifies areas with similar needs and offers economies of scale for intervention. The framework provides an opportunity to reduce historical stigmatization of neighborhoods labeled as “bad” or “lacking.” By balancing assessments of liabilities with the recognition of community assets, the framework celebrates neighborhood strengths and highlights resilience. Policymakers can use this framework to establish actionable targets for improving neighborhoods, incorporating health into neighborhoods, and comprehensive planning processes. This new framework fosters more equitable decision-making and ensures investments reflect community needs and strengths, creating healthier and more inclusive environments.

## Conclusion

Achieving health equity requires addressing environmental disparities. The UBN framework assesses health-supportive characteristics at the census tract level, revealing disparities that are often obscured by broader metrics. Focusing on detailed place-based data, it highlights nuanced differences across neighborhoods within cities and uncovers health inequities that aggregate data can mask. The current reliance on relative ranking and deficit-based models overlooks the impact of salutary factors, particularly in marginalized communities. Presenting negative and positive health determinants, the UBN framework leverages community strength, incorporates local voices, and clarifies community needs. The UBN can foster healthier and more equitable environments by uplifting all neighborhoods to these standards.

## Abbreviation Used

UBN: Universal Basic Neighborhood

## References

[1] World Health Organization, Commission on Social Determinants of Health. Closing the Gap in a Generation: Health Equity through Action on the Social Determinants of Health. World Health Organization: Geneva; 2008. Available from: https://www.who.int/publications/i/item/WHO-IER-CSDH-08.1 [Last accessed: January 15, 2025].

[2] University of Wisconsin Population Health Institute. County Health Rankings & Roadmaps 2024 National Findings Report. University of Wisconsin Population Health Institute: Madison, WI; 2024. Available from: https://www.countyhealthrankings.org/findings-and-insights/2024-national-findings-report [Last accessed: January 15, 2025].

[3] Braveman PA, Cubbin C, Egerter S, et al. Socioeconomic disparities in health in the United States: What the patterns tell us. Am J Public Health 2010;100(Suppl 1): S186–S196; doi: 10.2105/AJPH.2009.166082 20147693 PMC2837459

[4] County Health Rankings and Roadmaps. Jefferson County Kentucky Health Data. Available from: https://www.countyhealthrankings.org/health-data/kentucky/jefferson?year=2024 [Last accessed: January 15, 2025].

[5] Centers for Disease Control and Prevention (CDC). PLACES: Local Data for Better Health. Available from: https://www.cdc.gov/places/index.html [Last accessed: January 7, 2025].

[6] Fee E, Gonzalez AR. The history of health equity: Concept and vision. Divers Equal Health Care 2017;14(3):148–152.

[7] Farrer L, Marinetti C, Kuipers Cavaco Y, et al. Advocacy for health equity: A synthesis review. Milbank Q 2015;93(2):392–437; doi: 10.1111/1468-0009.12112 26044634 PMC4462882

[8] Harari L, Lee C. Intersectionality in quantitative health disparities research: A systematic review of challenges and limitations in empirical studies. Soc Sci Med 2021;277:113876; doi: 10.1016/j.socscimed.2021.113876 33866085 PMC8119321

[9] Shah SN, Russo ET, Earl TR, et al. Measuring and monitoring progress toward health equity: Local challenges for public health. Prev Chronic Dis 2014;11:E159; doi: 10.5888/pcd11.130440 25232746 PMC4170727

[10] Nelson ALH, Zanti S. A framework for centering racial equity throughout the administrative data life cycle. Int J Popul Data Sci 2020;5(1):1367; doi: 10.23889/ijpds.v3i5.1367 34007882 PMC8110889

[11] Griffith DM, Johnson J, Ellis KR, et al. Cultural context and a critical approach to eliminating health disparities. Ethn Dis 2010;20(1):71–76. 20178186

[12] Nieuwenhuijsen MJ, Dadvand P, Márquez S, et al. The evaluation of the 3-30-300 green space rule and mental health. Environ Res 2022;215(Pt 2):114387; doi: 10.1016/j.envres.2022.114387 36162472

[13] Takano T, Nakamura K. An analysis of health levels and various indicators of urban environments for Healthy Cities projects. J Epidemiol Community Health 2001;55(4):263–270; doi: 10.1136/jech.55.4.263 11238582 PMC1731876

[14] Trujillo MD, Plough A. Building a culture of health: A new framework and measures for health and health care in America. Soc Sci Med 2016;165:206–213; doi: 10.1016/j.socscimed.2016.06.043 27405727

[15] Healthy Communities Policy Guide Task Force. Healthy Communities Policy Guide. American Planning Association; 2023. Available from: https://planning.org/publications/document/9141726/ [Last accessed: January 15, 2025].

[16] Ricklin A, Shah S. Metrics for planning healthy communities. American Planning Association; 2017. Available from: https://www.planning.org/publications/document/9127204/ [Last accessed: January 16, 2025].

[17] Urban Land Institute. Building Healthy Places Toolkit: Strategies for Enhancing Health in the Built Environment. Urban Land Institute: Washington, DC; 2015.

[18] Ziafati Bafarasat A, Cheshmehzangi A, Ankowska A. A set of 99 healthy city indicators for application in urban planning and design. Sustainable Development 2023;31(3):1978–1989.

[19] Economist Intelligence Unit. The Global Livability Index. 2023. Available from: https://pages.eiu.com/rs/753-RIQ-438/images/Jun-Global-Liveability-Index-2023.pdf [Last accessed: January 24, 2025].

[20] World Health Organization (WHO). Healthy Cities: Effective Approach to a Rapidly Changing World. World Health Organization: Geneva; 2020. Available from: https://iris.who.int/handle/10665/331946 [Last accessed: January 15, 2025].

[21] Forsyth A. What is a healthy place? Models for cities and neighborhoods. Journal of Urban Design 2020;25(2):186–202; doi: 10.1080/13574809.2019.1662718

[22] Xu H, Jia Y, Sun Z, et al. Environmental pollution, a hidden culprit for health issues. Eco Environ Health 2022;1(1):31–45; doi: 10.1016/j.eehl.2022.04.003 38078200 PMC10702928

[23] Keith RJ, Hart JL, Bhatnagar A. Greenspaces and cardiovascular health. Circ Res 2024;134(9):1179–1196; doi: 10.1161/CIRCRESAHA.124.323583 38662868 PMC12208525

[24] Shaw M. Housing and public health. Annu Rev Public Health 2004;25(1):397–418. 15015927 10.1146/annurev.publhealth.25.101802.123036

[25] Glazener A, Sanchez K, Ramani T, et al. Fourteen pathways between urban transportation and health: A conceptual model and literature review. J Transp Health 2021;21:101070; doi: 10.1016/j.jth.2021.101070

